# Telomere lengths correlate with fitness but assortative mating by telomeres confers no benefit to fledgling recruitment

**DOI:** 10.1038/s41598-021-85068-x

**Published:** 2021-03-09

**Authors:** Rebecca C. Young, Alexander S. Kitaysky, Hugh M. Drummond

**Affiliations:** 1 Department of Biological Sciences, North Dakota State University, Stevens Hall 311, Fargo, ND USA; 2 Instituto de Ecología, Universidad Nacional Autónoma de México, Mexico City, Mexico; 3 Institute of Arctic Biology & Department of Biology and Wildlife, University of Alaska Fairbanks, 907 Koyukuk Dr, Fairbanks, AK 99709 USA; 4 Instituto de Ecología, Universidad Nacional Autónoma de México, Circuito Exterior S/N Anexo Jardín Botánico Exterior, Ciudad Universitaria, 04500 Mexico City, Mexico

**Keywords:** Ecophysiology, Molecular ecology

## Abstract

Assortative mating by telomere lengths has been observed in several bird species, and in some cases may increase fitness of individuals. Here we examined the relationship between telomere lengths of Blue-footed Booby (*Sula nebouxii*) mates, long-lived colonial seabirds with high annual divorce rates. We tested the hypothesis that interactions between maternal and paternal telomere lengths affect offspring and parental survival. We found that relative telomere lengths (RTL) were strongly positively correlated between members of a breeding pair. In addition, RTL of both parents interacted to predict fledgling recruitment, although fledglings with two very long-RTL parents performed only averagely. Telomere lengths also predicted adult survival: birds with long telomeres were more likely to survive, but birds whose mate had long telomeres were less likely to survive. Thus, having long telomeres benefits survival, while choosing a mate with long telomeres benefits reproductive output while penalizing survival. These patterns demonstrate that while a breeder's RTL predicts offspring quality, assortative mating by RTL does not enhance fitness, and a trade-off between different components of fitness may govern patterns of assortative mating by telomere length. They also illustrate how testing the adaptive value of only one parent’s telomere length on either survival or reproductive success alone may provide equivocal results.

## Introduction

Assortative mating, or a positive correlation between the phenotypes or genotypes of mates, is a common phenomenon among animal species, including assortative mating by size, age, and visual cues^1–3^, although the evidence for assortment by size has recently been questioned^4^. Yet both proximate and ultimate questions remain, including what types of traits assortative mating is observed in and whether assortative mating is adaptive. Assortative mating is most often measured for traits that the choosing mate can clearly assess (e.g., size), but it may also be present for physiological or even epigenetic traits (e.g., telomere length), in which case more work is needed on how animals may assess character states. To support a hypothesis of adaptive assortative mating, similarity between partners should correlate with fitness^3,5^.

Telomere length is an epigenetic trait that is often positively associated with individual quality, especially in birds^6,7^. Long telomeres have been linked to younger age^8–10^, increased reproductive output^11–13^ and higher survival^14,15^, although some studies find no relationship or a negative relationship between telomeres and reproduction^16–19^. Telomere length also reflects previous experiences of the environment, as mediated by the individual’s physiology^20,21^. Since telomeres can integrate epigenetic, experiential, and physiological information, an individual’s telomere length may predict its capacity or willingness to invest in reproduction as well as its pace-of-life decisions^22^, although this relationship may not be causal^23^. Furthermore, telomeres are heritable^24^. Hence, animals are expected to prefer mates with long telomeres, independent of which cues they use in mate assessment, and in species where both sexes exercise mate choice assortative mating for telomere length could result. Assortative mating by telomere length has been reported in several bird species in which it is unlikely to be confounded with age^25–28^, but assortment by telomere length should be assessed for confounds with age and methodological similarities^4^. It could also be a by-product of exposure to shared environments if pair bond durations are long^29^. However, the effect of such indirect mechanisms and shared environment will be weaker in species that frequently break pair bonds, as do many bird species including the Blue-footed Booby.

In this study we ask whether Blue-footed Boobies (*Sula nebouxii*, hereafter boobies) mate assortatively by telomere length or by age and whether telomere lengths and ages of boobies and their mates are related to fitness outcomes (adult survival and fledgling recruitment), a prerequisite for an adaptive function. Quality of the mate can influence parental investment patterns^30^ and therefore trade-offs between survival and current reproduction. Therefore the mate’s quality (potentially indicated by telomere length) may correlate with adult survival, although via a different mechanism from the relationship predicted for a bird’s own telomere length. We test for a correlation between telomere length and age, to determine if these measures can be separated and then probe the relative strength of mating assortment by telomere length versus age, and whether assortment by parental telomere lengths predicts fledgling recruitment. Boobies, long-lived seabirds that nest colonially on oceanic islands in the eastern tropical Pacific, lend themselves to asking these questions because of their mating system: approximately equal division of parental responsibilities between the sexes^31^ and mutual assessment of foot color as an indicator of quality^32,33^ imply mutual mate choice and high annual divorce rates result in partnerships between birds of different ages and a lack of correlation between age and pair bond duration^34^. Notably, in this species, parental ages affect fledgling viability (recruitment probability) interactively rather than additively: the partnership of an old parent with a young parent maximizes the recruitment probability of fledglings, but its effect on parental survival is unknown^35^. This interactive parental age effect may be related to a telomere length effect if telomere length correlates with age, and it demonstrates that non-additive parental effects are already present in this system. Therefore when testing the relationships between telomere length and fitness outcomes we included interactive as well as additive effects on fledgling recruitment.

## Results

### Telomere length and age

An individual’s age and, to a lesser degree, age^2^ predicted its RTL (Table 1; age: β =  − 0.111 ± 0.020, t =  − 5.58, *p* < 0.0001; age^2^: β = 0.0312 ± 0.014, t = 2.28, *p* = 0.0234), while sex did not (β = 0.00404 ± 0.027, t = 0.150, *p* = 0.88). The quadratic term for age was a shallow U-shape with a steeper slope in early life, indicating that telomere loss was highest in young birds and negligible in adulthood. When the same analysis was run without chicks (not shown) no age terms were retained, despite a sample reaching to 22 years old, confirming negligible cross-sectional telomere loss in adulthood (age: β =  − 0.018 ± 0.029, t =  − 0.62, *p* = 0.54). Because adults do not show strong cross-sectional loss of telomere length, we did not correct telomere length for age in subsequent analyses.

**Table 1:** Telomere length predicted by age and age^2^ in Blue-footed Boobies.

	Value	Std. Error	DF	t-value	*p* value
(Intercept)	0.832	0.0275	294	30.2	0.0000
Age	− 0.111	0.0199	294	− 5.58	0.0000
Age^2^	0.0312	0.0137	294	2.28	0.0234
Sex (male)	0.00404	0.0269	294	0.150	0.881
DNA quality	0.00130	0.00267	294	0.485	0.628

### Assortative mating by telomere length and age

**Figure 1:**
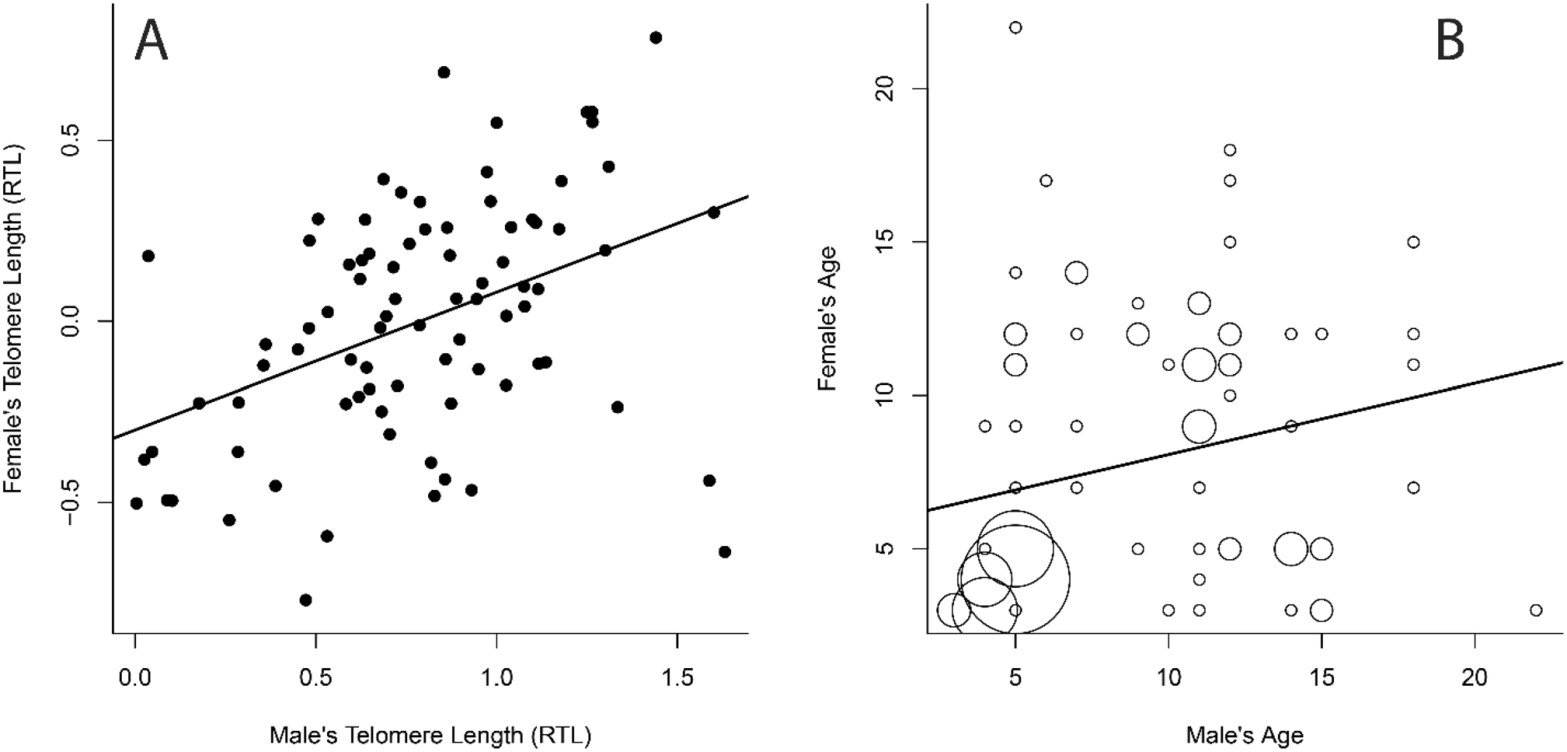
Assortative mating by telomere length (**A**) and age (**B**) in the blue-footed booby. In (**A**) each pair is represented by a point and the plotted trendline controls for other variables in the model (age and assay). In (**B**) the area of circle is proportional to the number of pairs with these ages. The assortment by telomere length is stronger than for age, despite many young pairs mated together.

After controlling for age and assay, male and female relative TL (RTL), measured by qPCR, were strongly positively related (male RTL: β = 0.48 ± 0.10 RTL units, t = 4.96, *p* < 0.0001; age: β = 0.028 ± 0.087 RTL units; t = 0.32, *p* = 0.75). Male and female ages were also positively correlated, but less strongly (β = 0.23 ± 0.11, t = 2.12, *p* = 0.037). A stronger relationship between parental RTLs (Fig. 1A versus B), showed stronger assortment by RTL than by age.

### Telomere lengths and adult survival

**Figure 2:**
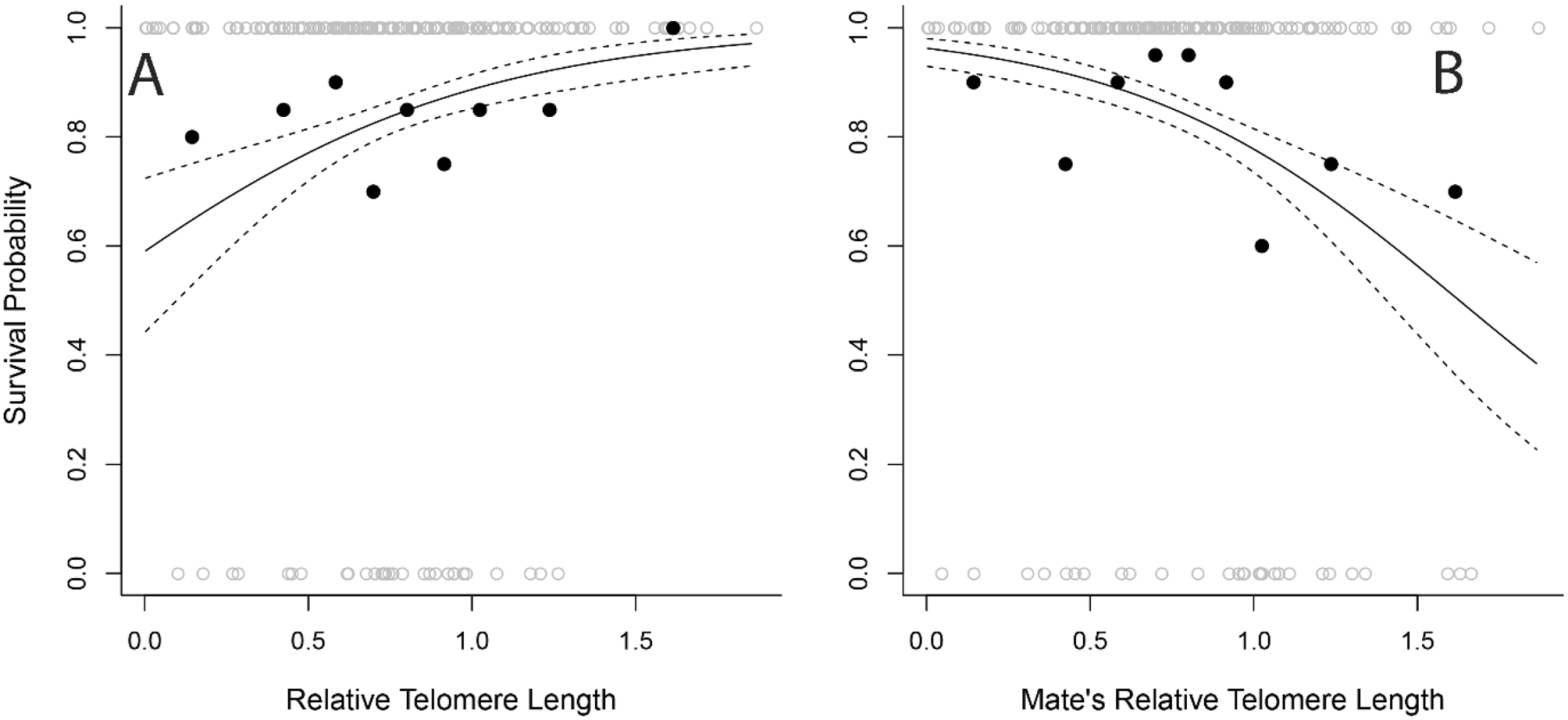
Adult survival based on (**A**) adult relative telomere length (RTL) and (**B**) RTL of the mate. Highest survival is for birds with long RTL whose mates have short RTL. Analysis was done on z-transformed values, but raw RTL’s are plotted here. Trendlines are based on model output with mean values for variables not pictured on the axes. Filled circles indicate mean survival and RTL (or mate’s RTL) for binned groups of aduls.

The model including partner’s RTL’s was a better fit than the one including ΔTL (increase in deviance = 8.2, *p* = 0.0042) and the ΔTL term (absolute difference between parental RTL values) was not significant (β = − 0.64 ± 0.78, z = − 0.82, *p* = 0.41). Adults with longer telomeres were more likely to survive, and adults whose mates had longer telomeres were less likely to survive (Table 2), but age and sex had no significant effect on survival (*p* > 0.19). Therefore, an individual survived better when its RTL was long and its mate’s RTL was short, but such pairings resulted in poor predicted survival for the mate (Fig. 2). Models run without the focal bird’s RTL retain a weakened negative effect of mate’s RTL (β = − 0.029 ± 0.016, z = − 1.84, *p* = 0.0663), but models run without the mate’s RTL do not retain the significant result for focal bird’s RTL (β = 0.00985 ± 0.016, z = 0.604, *p* = 0.546), indicating that the positive effect of the focal bird’s RTL on its own survival is largest when the mate’s RTL is having a stronger negative impact. In addition, both models increase the deviance, despite having more degrees of freedom (retaining only mate’s RTL: deviance increase = 5.4, *p* = 0.020; retaining only focal bird’s TL: deviance increase = 8.5, *p* = 0.0036).

**Table 2:** Predicting adult survival based on relative telomere length (RTL) of adult boobies and their mates using a logistic regression.

	Estimate	Std. Error	z value	*p* value
(Intercept)	2.45	0.515	475	< 0.0001
Age	− 0.0507	0.0443	− 1.14	0.252
RTL	0.052	0.023	2.20	0.0280
Sex (male)	− 0.568	0.437	− 1.30	0.193
Mate’s RTL	− 0.0597	0.0212	− 2.82	0.00488

Age included as a known influencer of survival. RTL variables are z-transformed. Residual deviance: 143.19 on 165 df.

### Telomere lengths and fledgling recruitment

**Figure 3:**
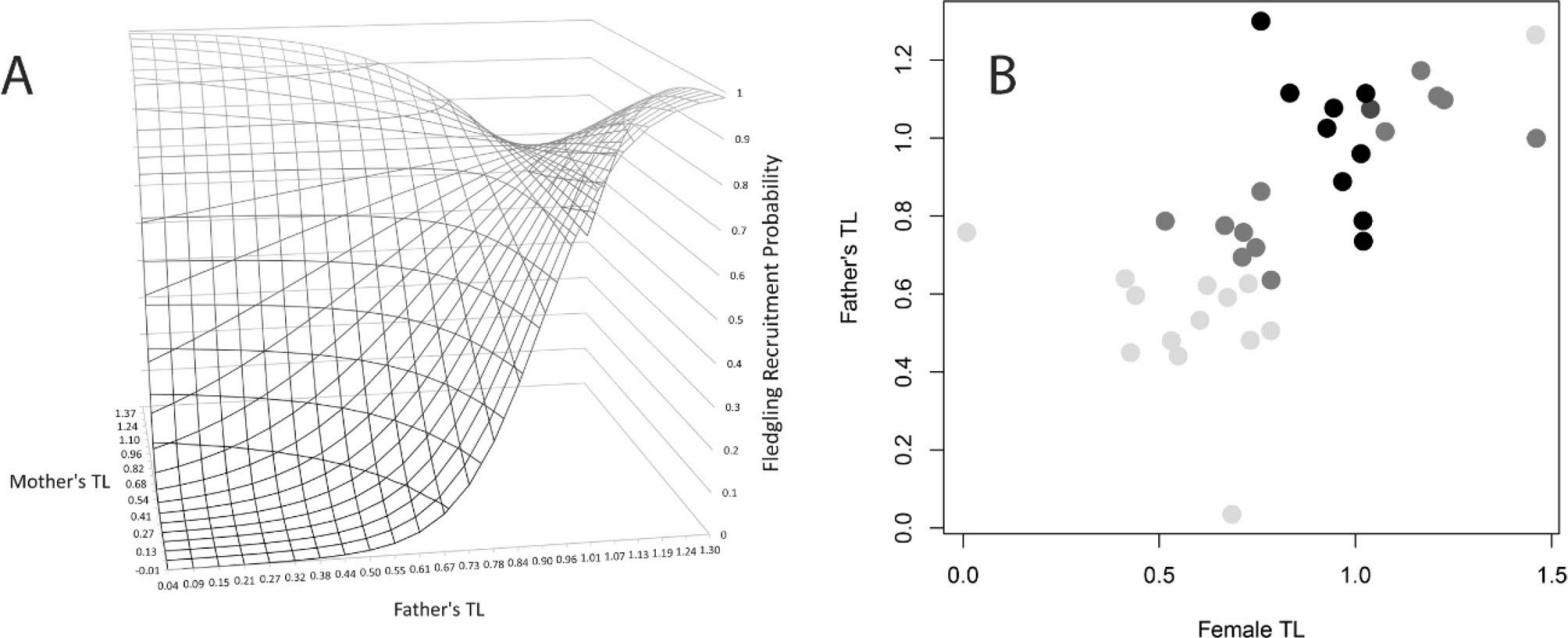
Interacting effect of parental relative telomere lengths (RTLs) on fledgling recruitment. (**A**) Predicted values of full model outcome showing an increase in fledgling recruitment as parental RTLs increase, but a diminishment again when both parents have very long RTLs. (**B**) Observed values of parental RTL and fledgling recruitment. Each point represents a mated pair, with the increasing darkness of the points indicating the increasing proportion of fledglings that recruited. Parents with similar and extreme RTLs have the lowest fledgling recruitment probabilities. Analysis was done on z-transformed values, but raw RTL’s are plotted here.

Parental RTLs interacted to predict fledgling recruitment (Table 3; β = − 1.08 ± 0.54, z = − 2.00, *p* = 0.045). The interaction shows a positive relationship with recruitment probability for most values of male and female RTLs, but when both partners had very long RTLs that combination depressed fledgling survival to some degree (Fig. 3). Removal of either parental RTL term significantly or nearly significantly increased deviance (mother’s RTL: Δ deviance = 5.4, *p* = 0.064; father’s RTL: Δ deviance = 6.9, *p* = 0.032). Sex, chick RTL, ΔTL, parental ages, and the interaction of parental ages were all statistically non-significant (all *p* > 0.27).

**Table 3:** Fledgling recruitment is predicted by an interaction of parental relative telomere lengths (RTL) using a logistic regression.

(A) Full model	Estimate	Std. Error	z value	*p* value
(Intercept)	− 11.8	4.77	− 2.47	0.0134
Mother’s RTL	10.9	6.24	1.74	0.0818
Father’s RTL	12.4	5.44	2.28	0.0227
Mother’s RTL: Father’s RTL	− 12.9	6.42	− 2.01	0.0450
Chick’s RTL	2.03	1.85	1.10	0.273
Sex (male)	− 0.611	0.617	− 0.991	0.322
Mother’s age	0.0407	0.132	0.308	0.758
Father’s age	− 0.0279	0.114	− 0.243	0.808
Mother’s age: Father’s age	0.00356	0.0129	0.276	0.783
ΔTL	− 0.854	2.77	− 0.308	0.758

ΔTL is the absolute difference between mates’ telomere lengths, as a proxy for assortative mating. Residual deviance: 67.2 on 50 df.

## Discussion

This study is the first to explore assortative mating by telomere length in relation to two components of fitness: adult survival and fledgling recruitment. Assortment itself was not linked directly to fitness outcomes, but aspects of parental mating pattern were associated with both their own survival, that of their mate, and the recruitment of fledglings. Thus, telomere length has long-term effects on fitness that likely drive life history patterns and reproductive investment decisions, but such effects may be complex and interactive rather than additive. Exploration of each species’ biology will be necessary to determine how individuals assess mate quality, especially in relation to telomere length, and how trade-offs are managed to result in increased fitness.

Assortative mating by telomere length has been observed in barn swallows (*Hirundo rustica*)^28^, tree swallows (*Tachycineta bicolor*)^26^, bluethroats (*Luscinia svecica*)^27^, and king penguins (*Aptenodytes patagonicus*)^25^, although it was absent in blue tits (*Cyanistes caeruleus*)^17^. In tree swallows, adults’ telomere lengths did not predict fitness, but pairs with the most disassortative telomere lengths had lower reproductive success and survival than assortatively mated pairs^26^. Effects of assortative mating by telomere length on fitness were not tested in the other species that mate assortatively by telomere lengths, but telomeres were associated with fitness in king penguins^25^ and with both fitness and a pigment-based ornament in barn swallows^12^. In comparison, here we observed assortative mating in boobies and relationships between RTL and adult and offspring survival but no direct effect of parental RTL similarity on either of those measures.

In our analyses of both personal survival and offspring survival, individuals with short telomeres had the lowest predicted outcomes, demonstrating a strong association in blue-footed boobies between RTL and individual quality or condition. In this booby the two sexes share all components of nest site choice, clutch and brood care almost equally^31^, and mutual mate choice is mediated by a process of extensive visual and auditory displaying by male and female, including the sky-pointing display involving extension of inverted wings and the parading display involving exaggerated foot-lifting^36^. During parading the foot webs are conspicuous, and tests of female responsiveness to male courtship have revealed that brighter webs, associated with better nutrition, younger age, and potentially lower oxidative damage levels^37–39^, elicit enhanced courtship and increased investment in eggs. Male responses to female webs also increase with their brightness^33^. Hence, assortment into pairs with similar RTLs is consistent with mutual choice of partners of high quality or condition, signalled by displays, and there is some evidence for assortative mating by foot colour^40^. All these foot colour covariates are potential telomere shortening factors^21,41,42^. In species such as the blue-footed booby whose RTLs are correlated with individual quality or condition, mate choice is likely to be based not directly on RTL, which a bird cannot assess, but on morphological, behavioural or physiological traits, such as web colour, that reliably index individual quality. In barn swallows, plumage colour is hypothesized to cue assortative mating by telomeres, where ventral plumage colour correlates with telomere length and also individual quality^12^. In common yellowthroats (*Geothlypis trichas*), reproductive success is correlated to both telomere length and colour as well^43^. Whether RTL is correlated with foot web brightness or any other aspect of boobies’ displays remains to be tested.

Surprisingly, a booby´s survival after the current reproductive season increases with the shortness of its mate’s RTL, implying that, independent of its own RTL, a booby of either sex can obtain a postponed benefit by mating with an individual of low quality. Indeed, in zebra finches (*Taeniopygia guttata*) there is some evidence of assortative mating by quality resulting from active preference for matched quality rather than a uniform preference for high quality among all choosers^44^. Benefits of mating with low-quality individuals could underlie such observations. Alternately, this pattern may be explained by the differential allocation hypothesis. Birds may increase their reproductive investment whenever they pair with a partner of higher quality, prioritizing investment in offspring that are likely to receive high quality genes or care in preference to the lower quality offspring expected from future pairings^45,46^. On this basis, low-quality boobies are likely to invest more on average in the current reproductive event than their mates, and high-quality boobies are likely to do the converse. Depending on their life history strategy, high-quality boobies could assign the investment they save in the current reproductive event to their own maintenance and survival or future reproduction, and the evidence of our analyses suggests that they choose maintenance and survival. Against this background, a booby of any RTL could maximize its own survival by choosing a mate with short telomeres and maximize the recruitment of its fledglings by choosing a mate with long telomeres. Confirmation of differential allocation in boobies will require measurement of how reproductive investment of both partners varies with their own and their mate’s quality^45^.

Despite the RTL’s of males and females affecting positively the recruitment of their offspring, indicating that boobies of high quality may produce high quality offspring by conferring better genes or parental care on them, fledglings whose parents both had very long RTLs had only an average probability of recruiting. This puzzling result is unlikely to be due to poor genetic quality of the fledglings if, as we have inferred, their parents are probably of very high quality. We suggest that it may be due to mutual downregulation of parental care contributions. Conceivably, when two very high quality adults breed together each one reduces its own investment in an attempt to draw compensatory investment by the other, and the conflict between them ends in a compromise in which both invest only moderately. Sexual conflict over investment in offspring has been demonstrated to potentially reduce offspring quality in simultaneously caring parents^47,48^. A similar explanation could account for the poor quality of booby fledglings produced by two very old mates^35^. Members of older age classes are generally expected to be of higher quality and provide better genes to offspring^49,50^, and indeed quality of booby fledglings tends to increase with mothers’ and fathers’ ages. However, pairs of very old boobies produce fledglings with low probability of recruiting, possibly because each parent downregulates its investment in response to the other’s age. In the present study we did not find any effect of parental ages on fledgling recruitment. Drummond and Rodríguez^35^ found weak effects when they analysed a sample of 3,361 fledglings, so it is unsurprising that our sample of 61 fledglings failed to confirm their result. It is also of note that the model structure where two linear terms interact forces any cross-section of the resulting surface to be linear as well, however, the underlying reality may be more non-linear. Perhaps all extreme pairings of RTL (very short-very long, very short-very short, very long-very long) are of lowered quality, while pairs of birds with more central RTL are the most successful. This would be a surface with a nadir in all four corners, with a dome in the middle. A model approach in which non-linear interaction terms are modelled (e.g., mother’s RTL^2^ x father’s RTL^2^) could reflect such a reality but would require more data than were available in this analysis.

This study of a long-lived seabird links telomere of a bird and its mate to adult survival and fledgling viability and demonstrates assortative mating by RTL. It also argues that when assessing fitness patterns that underlie behaviours like assortative mating it may be necessary to consider life history trade-offs between current and future reproduction, as well as the ways that parental investment decisions can vary with parents’ interactions and their respective qualities. Further work should focus on the relationships between telomeres and phenotypic traits used in mate choice and investment decisions, and analyse more complete measures of fitness (e.g., lifetime reproductive success and the costs or benefits of mate switching).

## Methods

### Study system and sample collection

Blood samples were collected during the peak chick-rearing period (February–May) in 2011 from adult and young blue-footed boobies on Isla Isabel, Nayarit, Mexico^51^. The study areas of this colony have been annually monitored for over 30 years, including checks on breeder identities and nest contents every 3–6 days over a five-month period and banding of fledglings^52^. Most breeders in study areas were of known age and reproductive history: in 2011 87% of observed adults were previously banded. Boobies lay clutches of 1–3 eggs and provide biparental care. Their reproductive output over the lifespan follows a typical inverted U pattern, with production of fledglings peaking in middle age (8–12 years old)^53^. Boobies in this population show strong lifetime philopatry: recruits first nest within 40 m of their natal nest site and thereafter nest close to their own first nest^54^. Only 0.12% of birds banded as fledglings have been recorded breeding on a different island^54^.

Samples were taken from families where at least one chick had survived to day 10, thus excluding nests with early mortality (which were difficult to capture due to logistics). Two chicks that died before fledging were excluded from analysis of fledgling recruitment to avoid confounding pre-fledging and post-fledging mortality. In addition, we preferentially sampled families with old parents, which are rare in the population, so the sample includes the range of available ages (this sample: mean ± SD adult age: 8.0 ± 4.5 years, range 3–22). Only 11.7% of the population survives to age 16^51^, and 25 is the oldest observed breeding age for males and females (H. Drummond, unpublished data).

Recruitment of a fledgling was scored (yes/no) if it ever nested in the focal area between 2012 and 2017. Most birds recruit between two and six years old^55^, so fledglings that had not recruited by 2017 likely did not survive to breed. Similarly, adults were scored for survival or non-survival from 2012–2017. Adult birds not re-sighted by 2017 were counted as “not survived”. All other patterns of presence/absence were scored as “survived”. This yields a conservative estimate of mortality, as some birds seen in 2012 or 2013 and then not seen again during four consecutive years may in fact be dead.

### Telomere analysis

Blood was taken from the wing and stored at room temperature in a buffer (1.0 M Tris: 0.5 M EDTA: 5.0 M NaCl: 10% SDS)^56^. Blood was always < 1 mL or < 1% of bird body mass, whichever was lower, which is consistent with the < 2% recommendation of the Ornithological Council^57^. DNA was extracted in 2017 and 2018 using DNeasy Blood and Tissue Kit (Qiagen), and stored in elution buffer at − 80 °C until qPCR. Some samples scored low on DNA quality measures (260/280 ratio below 1.8 or 260/230 ratio below 2), so DNA quality (the 260/230 ratio) was initially included in all models as a predictor, although it was never significant. It has therefore been removed from models and is not reported further. Adults (and chicks that recruited) were sexed by their adult voices (males whistle and females grunt). Chicks who fledged but never recruited were genetically sexed using primers from Merkling, et al.^58^. This study was carried out following the Animal Behavior Society's Guidelines for the Use of Animals in Research and the Ornithological Council’s Guidelines^57^ and in accordance with all Mexican legal requirements for research in national parks and animal conservation and welfare, in addition the NDSU IACUC provided a post-hoc review of protocols and found no animal welfare concerns. The study species is not classified as endangered or protected by norm 059–2010 of the Secretaría del Medioambiente y Recursos Naturales (SEMARNAT), and monitoring and sampling were done under SEMARNAT review and permissions (permit SGPA/DGVS/08333/10).

Relative telomere length (RTL) was measured using quantitative real-time PCR. This technique provides telomere quantity as a ratio of telomeric DNA to quantity of a single-copy reference gene (here GAPDH), but it does not provide lengths in base pairs. Protocols were adapted from Cawthon^59^, with primers from Foote^60^, where they were previously used for boobies. Reactions for telomere primers and reference gene primers took place on different plates, as the PCR protocols differed. The qPCR protocol for the reference gene (GAPDH) was 2 min at 50 °C, then 10 min at 95 °C, followed by 40 cycles of 15 s at 95 °C and 1 min at 60 °C. For the telomere reaction the protocol was 2 min at 50 °C, then 10 min at 95 °C, followed by 25 cycles of 15 s at 95 °C, 1 min at 53 °C, and 5.5 min at 72 °C.

Samples were run in triplicate on each plate, and coefficient of variation was calculated as (range/mean) which is more appropriate for small sample sizes (e.g., triplicate). Mean intraplate CV’s were 2.4% for GAPDH and 3.6% for telomeres. If a sample’s CV exceeded 10% the sample was excluded from analysis. Only one sample was thus excluded, due to a variable GAPDH reaction. Repeatability was assessed using an intra-class correlation coefficient and was quite high (ICC = 0.77)^61^. A golden sample was run on each plate and results were standardized to this sample, and family members were grouped on the same plates where possible. This should reduce sources of methodological error but may also have the effect of making family members appear artificially more similar than in reality. To avoid this bias, we have accounted for plate variation by including assay as a fixed effect. Primer efficiencies, calculated from a standard curve, were 100% and 110% for the GAPDH and for telomeres, respectively. The high efficiency of the telomere reaction is due to a long elongation step, which was necessary to generate products with clean dissociation curves. Despite these long elongation times, the NTC did not reach the threshold, indicating a lack of primer dimer contamination. Since efficiencies differed, we used the Pfaffl^62^ method of T/S calculation, which corrects for primer efficiency. For each reaction, the primer efficiency is raised to the power of the difference between the CT values of the control and the sample. The final ratio is a sample's target value divided by that of the reference gene. PCR product of the telomere reaction was confirmed to be telomeric DNA by running several randomly chosen samples on an agarose gel and probing with a ^32^P-labeled telomere oligo.

### Statistical analysis

Data were analysed using linear models (or logistic models for recruitment and survival analyses) in R^63^ using package nlme^64^. QPCR assay was included as a fixed effect to control for inter-plate variability. For all model families, collinearity of predictor variables was assessed with variance inflation factors (VIF). For every model, all VIF were < 2.44, indicating acceptable levels of collinearity. Conformation to parametric assumptions was assessed using residual plots.

There were four main analyses, testing (a) whether age and RTL were negatively related, (b) whether parents mated assortatively by RTL or age; (c) whether adult survival was related to RTL, mate’s RTL, or similarity between pairs (ΔTL, absolute difference between parental RTL values as a proxy for assortative mating); and (d) whether fledgling recruitment (reproductive success) was related to parental RTLs, their interactions, or ΔTL. Except for the assortative mating analysis, all RTL values were z-transformed to make them more comparable across studies^65^.

To test the relationship between age and telomere length we used 109 adult females (3–22 years old), 110 adult males (3–22 years old), 106 female chicks, and 110 male chicks. The model also included sex, age, and age^2^. A quadratic term was considered as often telomere loss is highest early in life^10,60^. Assortative mating by RTL and age was tested in 83 pairs for which RTL was analysed in the same assay (qPCR plate). The male value was used to predict the female value in both, and the RTL assortment model also controlled for age and assay. All continuous variables in the tests of assortment were scaled to make effect sizes comparable between analyses.

The effect of RTL on adult survival was tested using 170 birds from 85 pairs for whom RTLs were known, with predictors including RTL, mate’s RTL sex, and age. We also compared a model that included ΔTL rather than the partner’s RTL, to explicitly test the degree of assortment. Of these 170 adults, 112 were re-sighted in subsequent years (79%). Inclusion of RTL terms was tested using analysis of deviance. To test effects of parental RTL and fledgling RTL on fledgling recruitment we used 61 chicks from 36 nests for whom ages and RTL of both parents were available, including sex, maternal and paternal ages and their interaction, and maternal and paternal RTL, their interaction, and ΔTL. Chick’s RTL was included as this inherited value may better approximate the chick’s quality and survival probability. Despite correlations between chick and parental values, the VIF indicate no inflation of variance in estimates. Of these 61 chicks, 27 recruited (44%), a result comparable to the larger dataset (not analysed due to missing data) wherein 99 of 221 chicks recruited (44%). There were not enough degrees of freedom to include quadratic terms for parental ages or RTLs. A random effect controlling for nest would be appropriate, but resulted in a model with a singular fit, thus the model was fit without this term.

## Data Availability

Data will be submitted to DRYAD with no embargo upon acceptance.
